# Acute Respiratory Inflammation in Children and Black Carbon in Ambient Air before and during the 2008 Beijing Olympics

**DOI:** 10.1289/ehp.1103461

**Published:** 2011-06-03

**Authors:** Weiwei Lin, Wei Huang, Tong Zhu, Min Hu, Bert Brunekreef, Yuanhang Zhang, Xingang Liu, Hong Cheng, Ulrike Gehring, Chengcai Li, Xiaoyan Tang

**Affiliations:** 1 State Key Laboratory of Environmental Simulation and Pollution Control, College of Environmental Sciences and Engineering and Centre for Environment and Health, Peking University, Beijing, China; 2 Institute for Risk Assessment Sciences, Universiteit Utrecht, Utrecht, the Netherlands; 3 Department of Atmospheric and Oceanic Sciences, School of Physics, and Laboratory for Climate and Ocean-Atmosphere Studies, Peking University, Beijing, China

**Keywords:** air pollution intervention, black carbon, nitric oxide, PM_2.5_, respiratory inflammation, schoolchildren

## Abstract

Background: Epidemiologic evidence for a causative association between black carbon (BC) and health outcomes is limited.

Objectives: We estimated associations and exposure–response relationships between acute respiratory inflammation in schoolchildren and concentrations of BC and particulate matter with an aerodynamic diameter of ≤ 2.5 μm (PM_2.5_) in ambient air before and during the air pollution intervention for the 2008 Beijing Olympics.

Methods: We measured exhaled nitric oxide (eNO) as an acute respiratory inflammation biomarker and hourly mean air pollutant concentrations to estimate BC and PM_2.5_ exposure. We used 1,581 valid observations of 36 subjects over five visits in 2 years to estimate associations of eNO with BC and PM_2.5_ according to generalized estimating equations with polynomial distributed-lag models, controlling for body mass index, asthma, temperature, and relative humidity. We also assessed the relative importance of BC and PM_2.5_ with two-pollutant models.

Results: Air pollution concentrations and eNO were clearly lower during the 2008 Olympics. BC and PM_2.5_ concentrations averaged over 0–24 hr were strongly associated with eNO, which increased by 16.6% [95% confidence interval (CI), 14.1–19.2%] and 18.7% (95% CI, 15.0–22.5%) per interquartile range (IQR) increase in BC (4.0 μg/m^3^) and PM_2.5_ (149 μg/m^3^), respectively. In the two-pollutant model, estimated effects of BC were robust, but associations between PM_2.5_ and eNO decreased with adjustment for BC. We found that eNO was associated with IQR increases in hourly BC concentrations up to 10 hr after exposure, consistent with effects primarily in the first hours after exposure.

Conclusions: Recent exposure to BC was associated with acute respiratory inflammation in schoolchildren in Beijing. Lower air pollution levels during the 2008 Olympics also were associated with reduced eNO.

A number of epidemiologic studies have shown that urban particulate matter (PM) is associated with pulmonary inflammation and increased respiratory symptoms, deterioration of lung function, increased hospital admissions, and mortality from inflammatory lung injury (Pope and Dockery 2006; Smith et al. 2009; Steerenberg et al. 2001; Suglia et al. 2008). PM is a complex mixture of inorganic and organic chemical components; identifying the components that produce adverse health effects is crucial to the implementation of efficient abatement strategies to improve air quality. Black carbon (BC), formed from incomplete combustion of fossil fuels and biomass, is an important component of PM. *In vitro* and *in vivo* studies have shown that BC stimulates reactive oxygen species production and cytokine-mediated inflammation (Alessandrini et al. 2009; Soto et al. 2008), which may play major roles in adverse health effects related to the exacerbation of cardiopulmonary disease (Pope and Dockery 2006). Epidemiologic studies using BC as an indicator of traffic-related air pollution suggest that it can increase the risk of acute respiratory inflammation in elderly subjects with asthma (Jansen et al. 2005), decrease lung function (Suglia et al. 2008), and decrease antioxidant activity and induce systemic inflammation and platelet formation in subjects with coronary artery disease (Delfino et al. 2008). However, few epidemiologic studies have examined BC as a potential cause of respiratory acute inflammation in children. Fischer et al. (2002) and Steerenberg et al. (2001) reported increases in airway inflammatory markers in schoolchildren exposed to black smoke (BS), and Delfino et al. (2006) reported a robust association between elemental carbon (EC) and airway inflammation in asthmatic children. BC is highly correlated to EC (Park et al. 2006) and BS (Quincey 2007) but has different absolute values, which implies that BC is a good surrogate for EC and BS. Although BC, BS, and EC are used interchangeably in health studies, they are defined by different instrumentation and data processing, and direct quantitative evidence for effects of BC on respiratory inflammation in children is scarce.

Mean annual concentrations of PM_10_ (PM with an aerodynamic diameter of ≤ 10 μm) in Beijing are consistently higher than recommended by World Health Organization air quality guidelines (WHO 2006), and daily BC concentrations up to 20 μg/m^3^ have been occasionally observed (Wang et al. 2005). To improve air quality in Beijing, especially during the 29th Olympics and Paralympics from 8 August to 17 September 2008, the Beijing municipal government implemented systematic long- and short-term emission control policies, which included relocating polluting factories, strictly enforcing vehicular emission standards (Euro 4 standard; Asian Development Bank 2003), reducing on-road private cars, and limiting diesel vehicle access (for details, see Kipen et al. 2010; Wang M et al. 2009). Measurements have shown significant reductions in concentrations of ambient pollutants such as PM and BC during the Olympic Games period (Wang M et al. 2009; Wang X et al. 2009). The unusually low level of pollutants that resulted from the air pollution intervention and favorable weather circumstances offered a unique opportunity to assess the acute health response to the reduced pollution levels during the Olympics.

Inflammation is one of the most critical biological pathways for PM-induced health effects (Schwarze et al. 2006). Exhaled nitric oxide (eNO) is a sensitive noninvasive biomarker of airway inflammation that has been used in many epidemiologic studies of the impact of air pollutants on healthy and asthmatic subjects (Jansen et al. 2005; Koenig et al. 2003; Mar et al. 2005). It is now recommended as a clinical surrogate marker for airway inflammation by the American Thoracic Society (ATS) and the European Respiratory Society (ERS) (Reddel et al. 2009).

To investigate the association between acute respiratory inflammation and short-term exposure to gaseous pollutants, PM, and BC, we conducted a 2-year panel study in 2007 and 2008, covering the periods before and during the Olympic Games air pollution intervention. We used eNO as a biomarker for acute respiratory inflammation and on-site monitoring of hourly mean ambient air concentrations of BC, PM_2.5_ (PM with aerodynamic diameter ≤ 2.5 μm), carbon monoxide (CO), sulfur dioxide (SO_2_), and nitrogen dioxide (NO_2_) as exposure metrics for the air pollutants. We estimated associations between eNO and air pollutant exposures, examined exposure–response relationships between eNO and BC and PM_2.5_ over large ranges of BC and PM_2.5_ concentrations, assessed the relative importance of BC and other pollutants with two-pollutant models, and estimated effects of lagged exposure to BC on eNO. Some of the results of these studies have been previously reported as abstracts in conference proceedings (Lin et al. 2009; Zhu et al. 2008).

## Materials and Methods

*Study site.* The study was conducted in an elementary school attached to Peking University [see Supplemental Material, Figure 1 (http://dx.doi.org/10.1289/ehp.1103461)], which is 178 m and 300 m away from two busy roads. A continuous air pollution monitoring station is located about 650 m southwest of the school. A comparison of the air pollutant concentrations between the monitoring station and the elementary school showed good agreement (see Supplemental Material, Figure 2).

**Figure 1:**
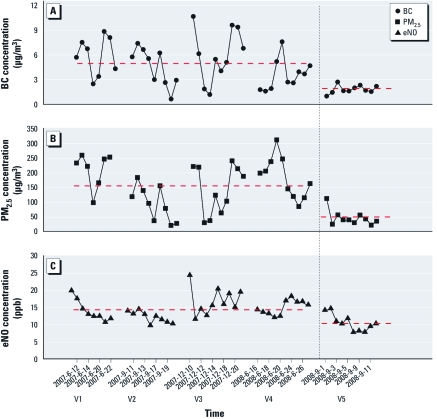
Average 0–24 hr concentrations for BC (*A*) and PM_2.5_ (*B*) and the corresponding averaged eNO concentrations for all subjects (*C*) during V1–V5. The dashed vertical line separates measurements taken before and during the Beijing Olympic Games air pollution intervention. Horizontal dashed lines indicate mean values before and after the intervention.

**Figure 2:**
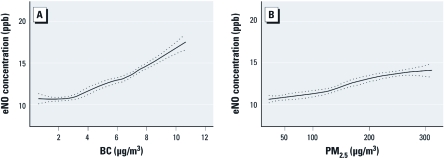
Relationship of BC (*A*) and PM_2.5_ (*B*) with eNO by the GEE model using nonparametric smoothed function smoothing (degree = 1, span = 0.65) with 95% bootstrap confidence intervals (dashed lines) after controlling for BMI, asthma, temperature, RH, and time.

*Participants.* At the beginning of the study, questionnaires were distributed to 734 students in grades 3 and 4 to obtain data about demographics, health status, and symptoms related to asthma, rhinitis, and eczema. Among the 437 students who returned the questionnaires, we recruited 8 students in grade 4 (6 boys, 2 girls) with doctor-diagnosed asthma. In addition, we randomly recruited 30 healthy children (12 boys, 18 girls) in grade 4 with no personal or family history of chronic respiratory disease or chronic inflammation. In total, we had 38 (18 boys, 20 girls) participants in the present study (mean age, 10.6 years).

The study included five observation periods, three in 2007 [visit 1 (V1), 11–22 June 2007; V2, 10–20 September 2007; V3, 10–21 December 2007] and two in 2008 (V4, 16–27 June 2008; V5, 1–12 September 2008). The last three observation periods each lasted 2 weeks and consisted of 10 weekday observations per subject. Only 8 days of observations were collected in June 2007 because of instrument malfunctions, and only 9 days of observations were collected in September 2007 because of a school holiday. During each period, we measured eNO for each subject every weekday (Monday through Friday), during the school lunch break. Data from one subject who withdrew after V2 in 2007, and one who withdrew before V5 in 2008 were not included in the analysis. Thus, we collected 1,581 valid observations from 36 subjects who each completed five visits.

The study protocol was approved by the Ethics Committee of the Centre of Health Sciences, Peking University. The guardians of all children gave their written consent for the children to participate in the research.

*eNO monitoring.* We collected the exhaled air samples from the subjects following the recommendations of the ATS/ERS (2005) for offline measurement. The exhaled air collection device was made of Teflon. A tube filled with activated carbon was used to filter out NO from the ambient air and was shown to remove up to 99.6% of NO in a laboratory simulation of the protocol used in the field. The device was equipped with a flow meter as a flow restrictor and a pressure indicator (Gong et al. 2006).

Before sampling, each subject was asked to put the mouthpiece of the device tightly in his or her mouth, inhale deeply, and then exhale to wash the “dead space” from the device. This procedure was repeated twice. To sample the exhaled air, the subjects were instructed to inhale to tidal capacity and then exhale into a 4-L air-sampling bag made of aluminium foil, with a constant flow of 150 L/hr and a positive pressure of 13 cm H_2_O (to close the soft palate and prevent nasal exhalation) (ATS/ERS 2005).

We measured eNO concentrations in the 4-L air-sampling bag within 4 hr with a chemiluminescence NO–NO_2_–NO_x_ (nitrogen oxides) analyzer (model 42i; ThermoScientific, Rockford, IL, USA), which had a detection limit of 0.4 ppb and a detection range of 0–100 ppb. The analyzer was calibrated every day with five concentrations (0–80 ppb) of NO (Beijing Huayuan Gas Chemical Industry Company Limited, Beijing, China) mixed in ultrahigh purity nitrogen (99.999%; Beijing Haikeyuanchang Practical Gas Co., Ltd.). We used a calibration curve to calculate the eNO concentrations in exhaled air of the subjects.

*Ambient air pollutant measurement.* Hourly averaged concentrations of PM_2.5_ (tapered element oscillating microbalance, RP1400a; ThermoScientific), BC (multiangle absorption photometer, model 5012; ThermoScientific), NO_x_, SO_2_, and CO (models 9841A, 9850A, 9830A; ECOTECH Pty Ltd., Knoxfield, Australia), and meteorologic parameters (Met One Instruments Inc., Grants Pass, OR, USA) were concurrently measured at the continuous monitoring site during V1–V5. The instruments for gaseous pollutants were automatically calibrated at 2300 hours every day. Additional details on methods and a comparison of PM_2.5_ and BC concentrations measured at the school and the fixed monitoring site are provided in the Supplemental Material (http://dx.doi.org/10.1289/ehp.1103461). In this study, we only report the results associated with outdoor concentrations of PM_2.5_, BC, CO, SO_2_, and NO_2_.

*Statistical analysis. t*-Tests were used to derive *p*-values for differences in mean air pollutants and eNO before and during the Olympic Games air quality intervention. Generalized estimating equations (GEE) (Liang and Zeger 1986), which correct for repeated measurements within subjects, were used to estimate associations between pollutants and eNO. To check the validity of the correlations between repeated measurements on the same subject, we used the “quasi-likelihood under the independence model criterion” (Pan 2001), which indicated that an autoregressive correlation matrix at lag 1 adequately fitted the data. The GEE calculations were conducted with the STATA statistical package (version 9.1; StataCorp LP, College Station, TX, USA) with robust standard errors, using the “force” option to allow for unequally spaced observations. The eNO data were transformed logarithmically. The model included adjustments for temperature, relative humidity (RH), body mass index (BMI), and asthma (yes or no). We also modeled interaction terms for asthma and each pollutant to evaluate differences in responses between nonasthmatic and asthmatic children.

To graphically analyze the exposure–response relationship between BC and predicted eNO, a nonparametric smoothed function (LOESS) was created with R statistical software (version 2.4.1; R Development Core Team, Vienna, Austria) (Cleveland 1979). The degree and smoothing parameter of the LOESS model were optimized according to the residual plot with a horizontal line fit to zero (Jacoby 2000). A LOESS model with a degree of 1 and a smoothing parameter of 0.65 was chosen for the analysis.

Two-pollutant models were used to investigate the stability of single-pollutant estimates and to check whether estimates for single pollutants might be biased because of confounding by other pollutants. We initially conducted the analysis including BC alone and PM_2.5_ alone and then considered the effects of SO_2_, NO_2_, and CO. These two-pollutant models provided the estimates of the individual effects of CO, SO_2_, and NO_2_ on eNO after adjustment for BC and PM_2.5_. All estimates were calculated with adjustment for temperature, RH, BMI, and asthma (yes or no).

To estimate effects of lagged BC exposures, we used a polynomial distributed-lag (PDL) model (Mar et al. 2005; Schwartz 2000). The PDL model allows effects at different lags to be estimated in the same model. To allow for autocorrelation, we adopted a third-degree PDL model with a maximum lag of 47 hr before eNO sampling based on the Akaike information criterion. The model equation can be written as follows:

ln(eNO*_i_*) = β_0_*X*_0_ + β_1_*X*_1_ + β_2_*X*_2_ + β_3_*X*_3_   + β_4_temperature + β_5_RH   + β_6_asthma*_i_* + β_7_BMI*_i_*,


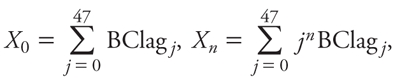


*n* = 1, 2, 3, [1]

where eNO*_i_* is the eNO concentration of the subject *i*; BClag*_j_* is the BC concentration at the *j*th hour before the eNO measurement; asthma*_i_* is the subject’s asthma status; and BMI*_i_* is the individual subject’s BMI.

The individual hourly estimate effects were obtained using the following equation:

BClag*_j_* = β_0_ + *j*β_1_ + *j*^2^β_2_ + *j*^3^β_3_. [2]

The PDL model was fit in STATA using commands developed by McDowell (2004).

## Results

Table 1 presents the characteristics of the 36 subjects. Their heights and weights were measured each year (June 2007 and June 2008); here we report measurements taken in 2007.

**Table 1:** The characteristics of the 36 subjects from the primary school.

Characteristic	Data
Age (years)	10.6 (9–12)
Weight (kg)*a*	37.8 (25.0–56.0)
Height (m)*a*	1.46 (1.3–1.6)
BMI (kg/m^2^)*a*	17.7 (13.7–23.9)
Sex	
Boy	17 (47)
Girl	19 (53)
Asthma	
Yes	8 (22)
No	28 (78)
Data are mean (range) or *n* (%). **a**Measured in June 2007, V1.	

During V1–V5, we observed a wide range of pollutant exposures, especially for BC and PM_2.5_ (Table 2). The 0–24 hr average concentration of BC ranged from 0.6 μg/m^3^ to 10.7 μg/m^3^; the 0–24 hr average concentration of PM_2.5_ ranged from 21.5 μg/m^3^ to 311.2 μg/m^3^ (Figure 1). During V5 (1–12 September 2008), which was during the Olympics/Paralympics air quality intervention from 20 July to 17 September (starting 2 weeks before the Olympics and ending after the Paralympics), average 0–24 hr concentrations of all pollutants were lower than in September 2007 (V2). Among V1–V5, 0–24 hr average BC and PM_2.5_ concentrations were the lowest for V5 (Table 2). Overall, mean BC and PM_2.5_ 0–24 hr concentrations were 64% and 70% lower, respectively, during the intervention period relative to all other time periods (*p* < 0.0001; Figure 1).

**Table 2:** Concentrations of air pollutants (0–24 hr) and eNO during V1–V5 in 2007 and 2008 (mean ± SD).

Variable	V1 (11–22 June 2007)	V2 (10–20 September 2007)	V3 (10–21 December 2007)	V4 (16–27 June 2008)	V5 (1–12 September 2008)
Environmental data										
PM_2.5_ (μg/m^3^)		212.0 ± 58.9		96.0 ± 58.7		144.4 ± 82.3		183.4 ± 70.1		46.4 ± 26.1
BC (μg/m^3^)		5.88 ± 2.3		4.52 ± 2.28		6.02 ± 3.20		3.57 ± 1.88		1.80 ± 0.48
CO (ppm)		1.28 ± 0.53		1.12 ± 0.46		2.85 ± 1.56		1.25 ± 0.46		0.88 ± 0.29
SO_2_ (ppb)		3.73 ± 2.68		9.09 ± 2.76		44.62 ± 21.90		1.15 ± 0.63		5.18 ± 3.55
NO_2_ (ppb)		24.31 ± 4.82		30.42 ± 9.97		45.29 ± 12.74		26.55 ± 6.96		25.85 ± 2.89
Temperature (°C)		28.0 ± 3.3		23.5 ± 4.0		1.1 ± 0.8		24.7 ± 2.5		25.6 ± 1.3
RH (%)		49.9 ± 14.7		66.1 ± 17.7		50.1 ± 12.9		72.0 ± 9.8		61.8 ± 12.0
eNO (ppb)										
All subjects		14.1 ± 7.3		12.2 ± 7.2		16.9 ± 6.9		15.0 ± 8.4		10.7 ± 7.9
Asthmatics		18.7 ± 10.2		15.6 ± 7.7		18.9 ± 6.8		20.7 ± 9.1		16.4 ± 11.1
Nonasthmatics		12.9 ± 5.7		11.2 ± 6.7		16.3 ± 6.8		13.4 ± 7.5		9.0 ± 5.8
Boys		13.2 ± 6.3		11.7 ± 6.0		17.2 ± 7.5		15.1 ± 8.3		10.7 ± 9.1
Girls		14.9 ± 8.0		12.6 ± 8.0		16.6 ± 6.3		14.9 ± 8.6		10.6 ± 6.8

Of the 1,581 observations, eNO concentrations varied from 1.3 to 71.5 ppb, with an overall mean ± SD concentration of 13.7 ± 7.9 ppb. Among all the subjects, the concentration of eNO decreased by about 27% (*p* < 0.0001) during the intervention relative to the other time periods (Figure 1, Table 2). Average eNO levels over V1–V5 were significantly higher in the asthmatic subgroup (18.0 ± 9.2 ppb) than in the nonasthmatic subgroup (12.5 ± 7.0 ppb; *p* < 0.05). We found no statistically significant differences between boys and girls.

Among all children combined, eNO was significantly and positively associated with interquartile range (IQR) increases in 0–24 hr PM_2.5_ (18.7% increase in eNO per 4.02-μg/m^3^ increase in PM_2.5_), BC (16.6% per 148.8 μg/m^3^), SO_2_ (8.1% per 11.38 ppb), CO (10.4% per 1.04 ppm), and NO_2_ (11.0% per 10.51 ppb) (Table 3). PM_2.5_ and BC were associated with greater increases in eNO per IQR increase than were the gaseous pollutants.

**Table 3:** Mean percent change (95% confidence interval) in eNO per IQR increase in each air pollutant over different exposure times.

Environmental exposure	All	Asthma	Nonasthma	*p*-Value*a*
PM_2.5_ (IQR = 148.8 μg/m^3^)								
0–24 hr		18.7 (15.0 to 22.5)		15.4 (5.9 to 25.7)		20.5 (16.5 to 24.5)		0.388
25–48 hr		4.4 (1.3 to 7.6)		–1.8 (–6.8 to 3.5)		7.2 (3.6 to 11.0)		0.043
0–48 hr		16.4 (11.8 to 21.2)		8.6 (–1.0 to 19.2)		19.7 (14.5 to 25.0)		0.150
BC (IQR = 4.02 μg/m^3^)								
0–24 hr		16.6 (14.1 to 19.2)		13.9 (7.3 to 20.8)		17.8 (15.4 to 20.3)		0.227
25–48 hr		9.3 (6.6 to 12.0)		4.4 (0.1 to 8.9)		10.9 (7.9 to 14.1)		0.084
0–48 hr		18.8 (15.4 to 22.2)		13.5 (5.7 to 21.8)		20.7 (17.3 to 24.1)		0.166
SO_2_ (IQR = 11.38 ppb)								
0–24 hr		8.1 (6.4 to 9.8)		6.3 (3.1 to 9.5)		8.7 (6.7 to 10.7)		0.137
25–48 hr		4.5 (3.4 to 5.6)		3.9 (1.6 to 6.2)		4.7 (3.4 to 6.0)		0.278
CO (IQR = 1.04 ppm)								
0–24 hr		10.4 (8.9 to 12.0)		10.1 (7.5 to 12.9)		10.8 (9.0 to 12.8)		0.270
25–48 hr		7.0 (5.0 to 9.0)		4.1 (1.7 to 6.7)		7.9 (5.5 to 10.3)		0.090
NO_2_ (IQR = 10.51 ppb)								
0–24 hr		11.0 (9.2 to 12.7)		11.5 (7.9 to 15.2)		10.8 (8.9 to 12.9)		0.399
25–48 hr		5.1 (3.4 to 6.9)		6.0 (2.1 to 10.0)		4.9 (3.0 to 6.9)		0.579
**a**Test of differences in percent change in eNO per increase in pollutant IQR between asthmatic and nonasthmatic children.								

eNO was also associated with IQR increases in PM_2.5_ and BC averaged over 25–48 hr (4.4% and 9.3%, respectively), but percent increases were much less than those associated with 0–24 hr averages (Table 3). BC averaged over 0–48 hr had the strongest association with eNO (18.8% eNO increase per BC IQR increase), but the estimated effect was likely dominated by the first 0–24 hr exposure.

Increases in eNO per IQR increases in PM_2.5_ and BC were somewhat smaller in asthmatic than in nonasthmatic children (Table 3), but differences were statistically significant (*p* < 0.05) only for an IQR increase in PM_2.5_ over the prior 25–48 hr.

Using the observed PM_2.5_ and BC concentrations and the predicted eNO value from GEE, we fit an exposure–response curve using the LOESS model. eNO increased almost linearly over the large range of BC (0.6–10.7 μg/m^3^) and PM_2.5_ (21.5–311.2 μg/m^3^) concentrations observed during the study (Figure 2).

**Figure 3:**
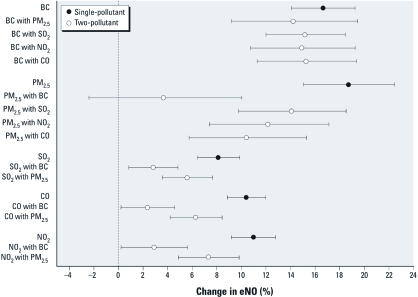
Single-pollutant and two-pollutant models for percent change in eNO per IQR change in pollutant of interest, using 0–24 hr average pollutant concentrations (mean and 95% confidence interval). All estimates are adjusted for BMI, asthma, temperature, and RH.

Adjusting for PM_2.5_ only slightly reduced the association between eNO per IQR increase in BC; similarly, adjusting for SO_2_, NO_2_, and CO only slightly reduced the association with BC (Figure 3). Adjusting for SO_2_, NO_2_, and CO in two-pollutant models did not change the PM_2.5_ effect estimate much, but the estimated percent change in eNO per IQR increase in PM_2.5_ was reduced to 3.6% (95% confidence interval, –2.4% to 10.0%) when adjusted for BC. Overall, BC had the most robust association with eNO when adjusted for confounding by other pollutants.

**Figure 4:**
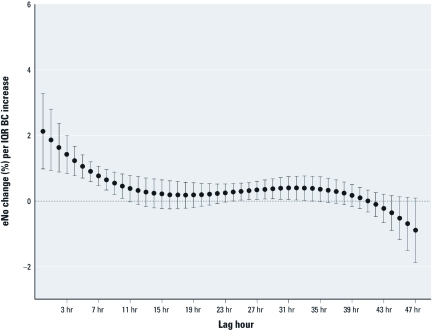
Mean (and 95% confidence interval) change in eNO per IQR increase (4.02 μg/m^3^) in BC. Estimates are calculated by a third-degree PDL model with a maximum lag of 47 hr before eNO sampling. All estimates are adjusted for BMI, asthma, temperature, and RH.

PDL model estimates for BC lagged up to 47 hr indicated that IQR increases in BC during the 10 hr before eNO measurements had the strongest associations with eNO (Figure 4).

## Discussion

Our findings suggest that reducing the concentrations of urban ambient pollutants during the 2008 Beijing Olympic Games air quality intervention was rapidly followed by a decrease in respiratory inflammation in children. Furthermore, we found a robust association between ambient BC and acute respiratory inflammation in children that persisted with adjustment for other pollutants. Previous studies also support effects of BC on airway inflammatory response. Jansen et al. (2005) estimated that a 1-μg/m^3^ increase in outdoor BC was associated with a 2.3-ppb increase in eNO in 16 older subjects with chronic respiratory diseases. Steerenberg et al. (2001) reported that a 50-μg/m^3^ increase in BS was associated with a 10-ppb increase in eNO at lag 1 day and a 9 ppb increase at lag 3 days in a study of 82 children 8–13 years of age. Fischer et al. (2002) found that eNO increased by 31.1% in association with a 10-μg/m^3^ increase in BS on the previous day in 68 children 10–11 years of age. Direct quantitative comparisons are not possible given differences in exposure metrics and study designs, but our findings are consistent with previous studies with regard to the association between BC and eNO and extend them by providing information on hourly lagged effects.

By taking advantage of the dramatic improvement in PM_2.5_ and its constituent BC during the 2008 Beijing Olympics, we were able to estimate the eNO exposure–response relationship based on a wide range of measured BC concentrations and show that it was more or less linear over the entire range. In our study, the BC IQR was 4.02 μg/m^3^, whereas in other studies IQRs were between 0.11 and 1.68 μg/m^3^ (Dales et al. 2008; Delfino et al. 2006; Jansen et al. 2005). In the context of the air pollution intervention during the 2008 Olympics, our panel study contributed to the existing evidence that drastic reduction in air pollutants over a relative short period of time can have immediate measurable health effects. Another intervention study in Dublin, Ireland (Clancy et al. 2002), which addressed at the effect on death by the ban on coal marketing, sale, and distribution within the city in 1990, concluded that control of carbonaceous particles resulted in decreasing respiratory and cardiovascular death rates and might have prolonged health benefits.

Our two-pollutant models have suggested that the estimated effect of PM_2.5_ on eNO was attributable mainly to confounding by BC (Figure 3). In contrast, the association between eNO and BC was not confounded by the other pollutants assessed. Overall, BC accounted for only 3% of the total PM_2.5_ mass (mean ratio of BC to PM_2.5_ concentration for each visit, listed in Table 2) in our study. It seems unlikely that all effects of PM_2.5_ on eNO could be due to this small mass fraction, and BC is highly correlated with other combustion products contributing to PM mass (e.g., polycyclic aromatic hydrocarbons) (Westerdahl et al. 2005). Delfino et al. (2006), in a study of schoolchildren, found a notable reduction in the estimated effect of PM_2.5_ on eNO after adjustment for EC, but, similar to our study, the association between EC and eNO was not confounded by PM_2.5_ or other gaseous pollutants. Schwartz et al. (2005) reported that BC had larger and more significant estimated effects on heart rate variability than did PM_2.5_ in an elderly population. Our study and others support the conclusion that BC may predominantly affect respiratory inflammation, in contrast with other pollutants.

Our findings also suggest that exposure to BC may have a lagged effect on eNO for up to 10 hr, in addition to an immediate effect. Previous studies have also supported rapid and delayed effects of PM on eNO in children, showing that exposures in the preceding 5–11 hr were associated with respiratory inflammation (Delfino et al. 2006; Mar et al. 2005).

Inflammation is a key factor in the pathogenesis of respiratory diseases (Mancini 2007), including those caused by ambient pollutants (Schwarze et al. 2006). A possible pathological mechanism for acute inflammation caused by BC has been suggested by toxicologic studies (Alessandrini et al. 2009; Soto et al. 2008). At the molecular level, inhaled carbonaceous particles have been shown to penetrate the epithelial cell lining, cause cell death, initiate cytotoxic responses, augment allergen-induced lipid peroxidation, and activate redox-sensitive transcription factors that trigger nuclear factor-κB–induced inflammation in the lung.

In the present study, BC and PM_2.5_ exposure appeared to have less of an impact on eNO in asthmatic children than in nonasthmatic children, but differences were not statistically significant, possibly because of limited power because of the small number of asthmatic children. The absence of effect modification by asthma has also been reported by Holguin et al. (2007). Their study of 95 asthmatic and 99 nonasthmatic children 6–12 years of age showed that estimated effects of traffic-related exposures and road density exposure were comparable between asthmatic and nonasthmatic children. They measured EC, PM_2.5_, and other traffic-related air pollutants in their study, with exposures somewhat similar to those our study. Our findings call for further investigation of asthmatic responses to BC using larger numbers of subjects.

## Conclusions

Short-term exposure to BC showed a positive monotonic dose–response relationship with acute respiratory inflammation in schoolchildren living in Beijing. This relationship was not confounded by PM_2.5_, which was not as strongly associated with eNO. The substantially lower air pollution levels during the 2008 Olympics were associated with reduced eNO.

## Supplemental Material

(180 KB) PDF
