# Supplementary material for: Acute Respiratory Inflammation in Children and Black Carbon in Ambient Air before and during the 2008 Beijing Olympics
Source: Environ Health Perspect. 2011 Jun 3;119(10):1507–12. doi: 10.1289/ehp.1103461 (PMC3230448; doi:10.1289/ehp.1103461)
Supplement: (180 KB) PDF [file ehp.1103461.s001.pdf]

Supplemental Material

Acute Respiratory Inflammation in Children and Black Carbon in Ambient Air Before and During the 2008 Beijing Olympics

Weiwei Lin, Tong Zhu, Wei Huang, Min Hu, Bert Brunekreef, Yuanhang Zhang, Xingang Liu, Hong Cheng, Ulrike Gehring, Chengcai Li and Xiaoyan Tang

Methods

Study site

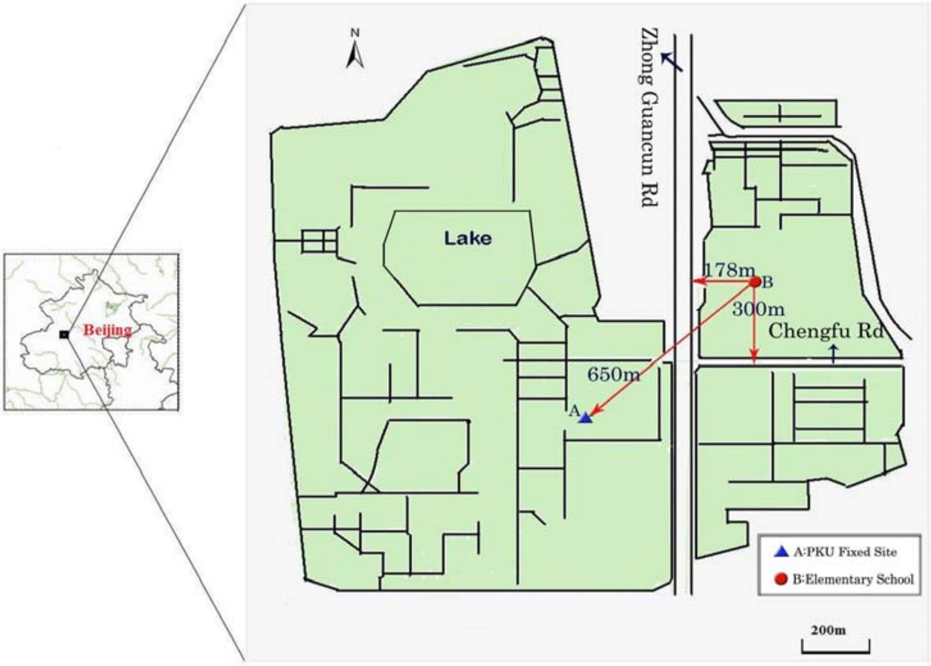

Supplemental Material, **Figure 1.** Location of the continuous air pollution monitoring site (A), the study site in the primary school attached to Peking University (B), and the main roads nearby.

In the children's panel study, we took advantage of a continuous fixed monitoring

station which is located about 650m away from the elementary school (Supplemental Material, Figure 1). In order to validate the representativeness of the pollutant concentration at this fixed site for the school exposures, we made comparison of pollutants, including black carbon in $PM_{2.5}$ (BC), black carbon in Total suspended particles (TBC) and $PM_{2.5}$. Figure 2 (Supplemental Material) shows the comparison of BC and $PM_{2.5}$ measured at the fixed monitoring station and TBC and $PM_{2.5}$ measured at the elementary school during the whole period of the study. The measurements of BC (multi-angle absorption photometer, model 5012, ThermoScientific, USA) and TBC (Aethalometer, model AE16, Magee Scientific, USA) were both based on optical method. $PM_{2.5}$ filter based samplers (Genie VSS5, Buck Inc, USA) were located at the elementary school as part of this special study to compare with $PM_{2.5}$ (tapered element oscillating microbalance, RP1400a, ThermoScientific) at the fixed station. The comparison included each measurement for which the pollutants were collected continuously at the fixed monitoring station during the pollutants measured at the elementary school. The correlation coefficients (R^2) between pollutants at these two sites were 0.93 and 0.56 respectively. The results, along with slope values close to 1 (1.5 for black carbon and 0.78 for $PM_{2.5}$), demonstrated that the BC and $PM_{2.5}$ measured at the fixed monitoring station were representative.

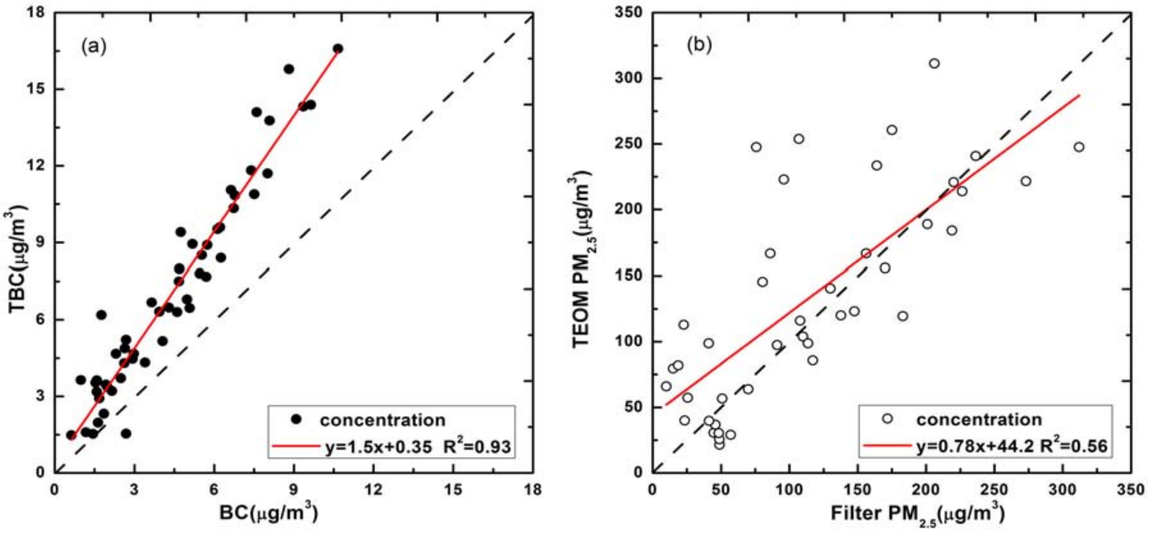

Supplemental Material, **Figure 2.** (a) Comparison of daily black carbon (BC) concentrations measured at the fixed monitoring station and total black carbon (TBC) concentrations measured at the elementary school during the study; (b) comparison of daily $PM_{2.5}$ (TEOM) concentrations measured at the fixed monitoring station and $PM_{2.5}$ (Filter) concentrations measured at the elementary school during the study. The solid red lines correspond to linear fit of measured data while the dash lines show the 1:1 ratio.
